# INFORMATIVE, DULL, INCOMPREHENSIBLE: SEEKING A COMMON VOCABULARY AROUND DATA IN LATER LIFE

**DOI:** 10.1093/geroni/igae098.3552

**Published:** 2024-12-31

**Authors:** Nicole Dalmer, Cal Biruk

**Affiliations:** McMaster University, Hamilton, Ontario, Canada; McMaster University, Hamilton, Ontario, Canada

## Abstract

With the domestication of digital devices into the everyday, this paper offers critical reflections from two, related studies that both sought to explore how older Canadians communicate their experiences and understandings about the digital technologies and related data they encounter and use. The first study, a virtual, qualitative survey with 70 older Canadians highlighted their understandings of data, and the second, a pilot interview study with 7 older adults in their homes explored how and where data circulates in their everyday lives. Not only do our daily activities and routines rely on data, our routines are often, in turn, converted into data through our many devices (Burgess et al., 2022). Living with data, however, is an experience that differs from person to person and from group to group. Given these differences, how might we communicate about data with different groups? In both studies, we sought to explore and document participants’ own diction, images, metaphors, and concepts they articulate when thinking with and about their own data; giving them a space to speak and share “their own data stories louder than those that are being told about them by others” or by other devices (Thorp, 2021, p. 19). In this paper, we offer our observations on the challenges and difficulties (and potential ways forward) in finding a common vocabulary in research relationships between participants and researchers to speak about data and to the many ways that data circulate in and around older adults’ everyday lives.

